# Adaptation to Preceding Acute Psychological Stress is Associated With Subsequent Stress Coping Levels via Corticoid Receptors

**DOI:** 10.31083/AP46061

**Published:** 2025-08-26

**Authors:** Yuta Aoto, Emi Kasama, Tohru Matsuki, Kenjiro Seki

**Affiliations:** ^1^Department of Pharmacology, School of Pharmaceutical Science, Ohu University, Koriyama, 963-8611 Fukushima, Japan; ^2^Department of Cellular Pathology, Institute for Developmental Research, Aichi Developmental Disability Center, Kasugai, 480-0392 Aichi, Japan

**Keywords:** fear, stress coping behavior, resilience, corticoid receptor, corticotropin-releasing hormone

## Abstract

**Objective:**

Hypothalamic‒pituitary‒adrenal axis response is essential for coping with acute stressors, while maladaptive stress coping may increase the risk of major depressive disorder. We previously demonstrated that behavioral patterns induced by prior psychological stress predict coping levels in response to future stressors. This study investigated whether activating corticotropin-releasing hormone (CRH) and corticosteroid receptors mediates psychological stress-induced coping behavior.

**Methods:**

Behavioral responses in mice exhibiting a fear response elicited by exposure to 2,5-dihydro-2,4,5-trimethylthiazoline (TMT), a synthetic component of fox feces, as preceding psychological stress, were assessed by measuring central zone entries in an open-field test. Time spent immobile during the tail suspension test was evaluated as a subsequent aversive stress-coping level. CRH overexpression was induced by adeno-associated virus injection (Hypo-CRH-OE) into the paraventricular hypothalamic nucleus. Dexamethasone (10 μg/kg, s.c.), a glucocorticoid receptor agonist, or fludrocortisone (5 mg/kg, s.c.), a mineralocorticoid receptor agonist was administered 30 min before behavioral tests.

**Results:**

Hypo-CRH-OE mice exhibited significantly higher plasma corticosterone levels than controls, without changes in baseline of locomotor activity or innate fear sensitivity. During TMT exposure, Hypo-CRH-OE mice showed lower central activity in the open-field test, accompanied by longer immobility time in the tail suspension test (TST), disrupting the correlation between these behaviors. A similar disruptive effect was observed in fludrocortisone-treated mice but not in dexamethasone-treated mice. Additionally, fludrocortisone, but not dexamethasone, prolonged immobility during the TST.

**Conclusions:**

Preceding psychological stress-induced behavioral patterns may predict coping levels through mineralocorticoid receptor activations offering a potential target for improving stress resilience and preventing depression.

## Main Points

1. The hypothalamic‒pituitary‒adrenal (HPA) axis activation plays a key role in acute stress adaptation and coping.

2. Psychological fear of stress may shape behavioral patterns that influence subsequent stress-coping levels.

3. Mineralocorticoid receptor activation influences acute stress adaptation and coping behavior.

4. Glucocorticoid receptors had minimal effects on stress-related adaptation and coping behaviors.

## 1. Introduction

Active coping is related to stress resilience [1], which can prevent the manifestation of depression-like phenotypes in response to stress [2]. Over the past decade, identifying the neurobiological mechanisms underlying resilience has become a crucial research focus to prevent the development of major depressive disorder (MDD). Active and passive coping strategies are particularly relevant under stressful conditions, where selecting an appropriate strategy determines the outcome [3]. Resilience is closely associated with active coping, defined as behavioral responses aimed at reducing the physical, psychological, or social harm caused by stress [1]. Passive coping behaviors in rodents, exemplified by helplessness [4], suggest that adopting passive coping strategies in response to psychological stress increases the risk of developing major depressive disorder (MDD). Despite its significance, no robust animal model has been established to study stress coping in relation to MDD risk, leaving the neurobiological mechanisms of stress maladaptation not fully understood. However, multiple genes influencing both resilience and vulnerability have been identified [5].

Stress-induced psychiatric disorders require a comprehensive understanding of interactions among endocrine function, neural networks, and adaptive coping responses [6]. Resilience is characterized by optimal hypothalamic‒pituitary‒adrenal (HPA) axis activity, which promotes active coping and adaptation to stress [6]. Dysregulation in the HPA axis, triggered by excessive secretion of cortisol in humans or corticosterone in rodents, increases the risk of MDD [7]. Studying the HPA axis’s role in resilience can provide insights into stress-coping levels [8]. Recent studies indicate that the glucocorticoids cortisol and corticosterone enhance resilience, enabling individuals to cope with threats and adversity [9]. Both cortisol and corticosterone activate mineralocorticoid receptors (MR) and glucocorticoid receptors (GR). The affinity of glucocorticoids for GRs is ten times lower; therefore, under baseline conditions, GRs are only partially bound to the corticoid receptor [10]. Consistent with the differing receptor binding affinities between MR and GR, chronic stress has been shown to result in a prolonged increase in glucocorticoids, which is linked to the onset of major depression and anxiety disorders [11]. Concurrently, higher MR expression in the hippocampus has been suggested to be involved in the brain’s adaptation to acute psychological stress [12]. This suggests that preventing MDD resulting from chronic psychological stress may require different coping strategies for acute stress mediated by MR and chronic stress-induced GR activation.

Understanding typical behavioral patterns against preceding stress is essential for identifying factors that reflect the coping levels in subsequent stress [13]. Predator odor stress inoculation, for example, has been proposed as an effective method to train animals to respond to severe stress [14]. In our previous study, mice subjected to 2,5-dihydro-2,4,5-trimethylthiazoline (TMT), a synthetic predator odor eliciting innate fear, exhibited a decrease in coping levels when subsequently subjected to tail suspension test (TST), an inescapable aversive stressor. However, mice that demonstrated a strong preference for the central zone in an open-field (OF) box during TMT exposure showed significantly greater resilience [13]. Therefore, we used this model to investigate the role of the HPA axis, along with MR and GR, in shaping behavioral patterns during fear stress, which reflect stress-coping behaviors during subsequent aversive stress.

## 2. Methods

### 2.1 Animals and Ethics

It has been reported that there is a sex difference in response to fear-related stress [15]. In addition, to minimize the effects of variations in stress responses across different phases of the estrous cycle and the influence of sex hormones [16], and to focus on the effect of corticotropin-releasing hormone, we used only male mice in the present study. Male C57BL/6J mice, aged 6–7 weeks, were purchased from CLEA Japan (Tokyo, Japan). Mice were housed at 25 ± 2 °C under a 12-h light/dark cycle (light: 08:00–20:00 h) with *ad libitum* access to food (#CE-2, CLEA Japan, Tokyo, Japan) and water. Groups of 4–5 mice were kept per cage. The study was approved by the Animal Care Committee of Ohu University (2021-14 and 2022-20) and adhered to and under the ARRIVE guidelines, all efforts were taken to minimize distress and use the minimum required number of animals. Behavioral tests were conducted between 11:00 and 16:00 h and analyzed by an independent investigator using ANY-maze software (v6.35; Muromachi Kikai, Tokyo, Japan). Dexamethasone (#D4438) was purchased from Tokyo Kasei (Tokyo, Japan), and fludrocortisone acetate (F6127-1G) was procured from Sigma-Aldrich (St. Louis, MO, USA).

### 2.2 Measurement of Plasma Corticosterone

Mice were anesthetized using medetomidine hydrochloride (0.3 mg/kg, Cat. No. 021-19001, Wako Pure Chemical Industries, Ltd., Tokyo, Japan), butorphanol tartrate (5 mg/kg, Cat. No. 135-17473, Wako Pure Chemical Industries, Ltd.), and midazolam (4 mg/kg, Product No. 21800AMX10357000, Sandoz, Ltd., Yamagata, Japan). Blood samples were collected via cardiac puncture using a 26-gauge syringe. Plasma was prepared with 1.0% citrate (Cat. No.58012-17, Kanto chemical Co. INC., Tokyo, Japan), 1.5 mg/mL EDTA-2Na (Cat. No. 15112-22, Nacalai tesque, Kyoto, Japan), and 12 U/mL heparin (Cat. No. 085-00134, FUJIFILM Wako Pure Chemical Corporation, Osaka, Japan) to prevent coagulation. The plasma was then centrifuged at 1200 ×g for 15 minutes at 4 °C, and the supernatants were stored at –80 °C for corticosterone measurement. Plasma corticosterone levels were quantified using an EIA kit (#YK240, Yanaihara Inst., Shizuoka, Japan). Serum samples were analyzed in duplicate, with a detection limit of 0.21 ng/mL. Standard curves were used, and absorbance at 450 nm was measured with a microplate reader (BioTek Instruments, Winooski, VT, USA) according to the manufacturer’s guidelines.

### 2.3 Exposure to TMT

We previously demonstrated that TMT exposure elicits a significant fear response in mice [13]. In this study, a total of 56 male C57BL/6J mice (8–12 weeks old) were used for the TMT-induced inescapable innate fear test. The experimental group consisted of 42 mice exposed to TMT, while the mineral oil (MO) control group included 14 mice.

One day before the test, the mice underwent a 15-minute habituation period in the TMT test box arena. Each mouse was placed in the center of an acrylic OF box (W: 294 mm × D: 294 mm × H: 297 mm), with the bottom and inner walls covered in non-reflective paper. The transparent plexiglass top allowed video recording using a webcam. A circular filter paper (D: 2 cm, Cat# 1001-025; Whatman GE Healthcare Life Science, Little Chalfont, UK) infused with 20 µL of TMT was placed in a corner of the odor exposure box.

The TMT odor was introduced 5 minutes after the mice were placed in the OF box, and fear-related behaviors were observed for 15 minutes. Three test boxes were prepared, with 3 to 6 mice tested per day to prevent reuse of the same box within an hour. Between experiments, the TMT odor was removed by treating each box with a 5% bleach solution for 30 minutes, followed by washing with detergent and water, and then wiping with 70% ethanol.

To ensure baseline behavior, we confirmed that the mice did not exhibit freezing behavior during a 5-minute OF test before TMT exposure.

### 2.4 Measurement of Freezing Behavior

We previously reported the measurement of freezing behavior during TMT exposure [13]. Herein, in this study, freezing behavior, described as the absence of all movement except for respiration [17], was used as a measure of fear in an acrylic box (294 × 294 × 297 mm) lit with 60 lux. The amount of time spent freezing, expressed as a percentage, was calculated for each mouse during the 10-minute period following TMT exposure, with TMT applied to the filter paper in the corner of the OF box. Freezing duration was analyzed in two 5-min bins: the first (6–10 min) and the second (11–15 min).

### 2.5 TST

We previously reported the detailed method of the TST [18]. In this study, the test was conducted during the light phase (13:00–16:00 h) of the light-dark cycle. Mice were individually suspended by the tail using adhesive tape, placed 2 cm from the tail tip, and attached to the ceiling of the test box, positioned 42 cm above the bench. The suspension lasted for 10 minutes, and behavior was recorded using a digital camera without the investigator present.

An independent investigator, blinded to group allocation, measured immobility time using ANY-maze software. Mobility was defined as any movement of the hind legs or other behaviors indicating an attempt to escape, excluding breathing. Immobility was defined as a motionless state lasting at least 2 seconds, with sensitivity set to 80%. After the TST, mice were returned to their home cages [18]. We previously reported that a decrease in active coping is typically observed during the first half of the 10-minute TST recording. However, differences in learned despair tend to emerge during the latter half of the test [13, 19]. Therefore, in this study, immobility time was measured separately for the first five minutes and the subsequent five minutes. Both durations were used as indices of passive coping behavior.

### 2.6 Statistics

Statistical analyses were performed using EZR (version 1.38, Jichi Medical University, Tochigi, Japan) [20] and BellCurve software (version 3.20, Social survey research information co., ltd., Tokyo, Japan) [21]. The Student’s *t*-test was used to compare groups, one-way ANOVA for single-factor comparisons, and two-way repeated measures ANOVA for multiple comparisons over time. Post hoc analysis was conducted using the Bonferroni test.

For correlation analyses, the normality of each set of behavioral measures was first assessed using the Shapiro-Wilk test. Pearson’s correlation coefficient was then calculated to examine relationships between variables. When conducting the Student’s *t*-test, homogeneity of variance was assessed using the F value, and Cohen’s d effect size was calculated to quantify the relative magnitude of differences between groups. Cohen’s *d* represents the standardized difference between two means, expressed in units of standard deviation.

Effect sizes were classified based on the absolute values of Cohen’s d, using the following thresholds: d ≤ 0.5 (small effect), 0.5 < d < 1.0 (moderate effect), 1.0 ≤ d < 1.5 (large effect), and d ≥ 1.5 (very large effect) [22]. An a priori power analysis was conducted to determine the appropriate sample size, incorporating an anticipated effect size (Cohen’s d > 1.5, representing a very large effect) and a predefined significance level (*p*) for the Student’s *t*-test [23]. Data are presented as mean ± standard error of the mean (SEM), with significance set at *p* < 0.05 (*) or *p* < 0.01 (**).

## 3. Results

### 3.1 Properties of Hypothalamic Corticotropin-Releasing Hormone (CRH) Overexpression Mice Before and During TMT Exposure

**Fig. 1.**
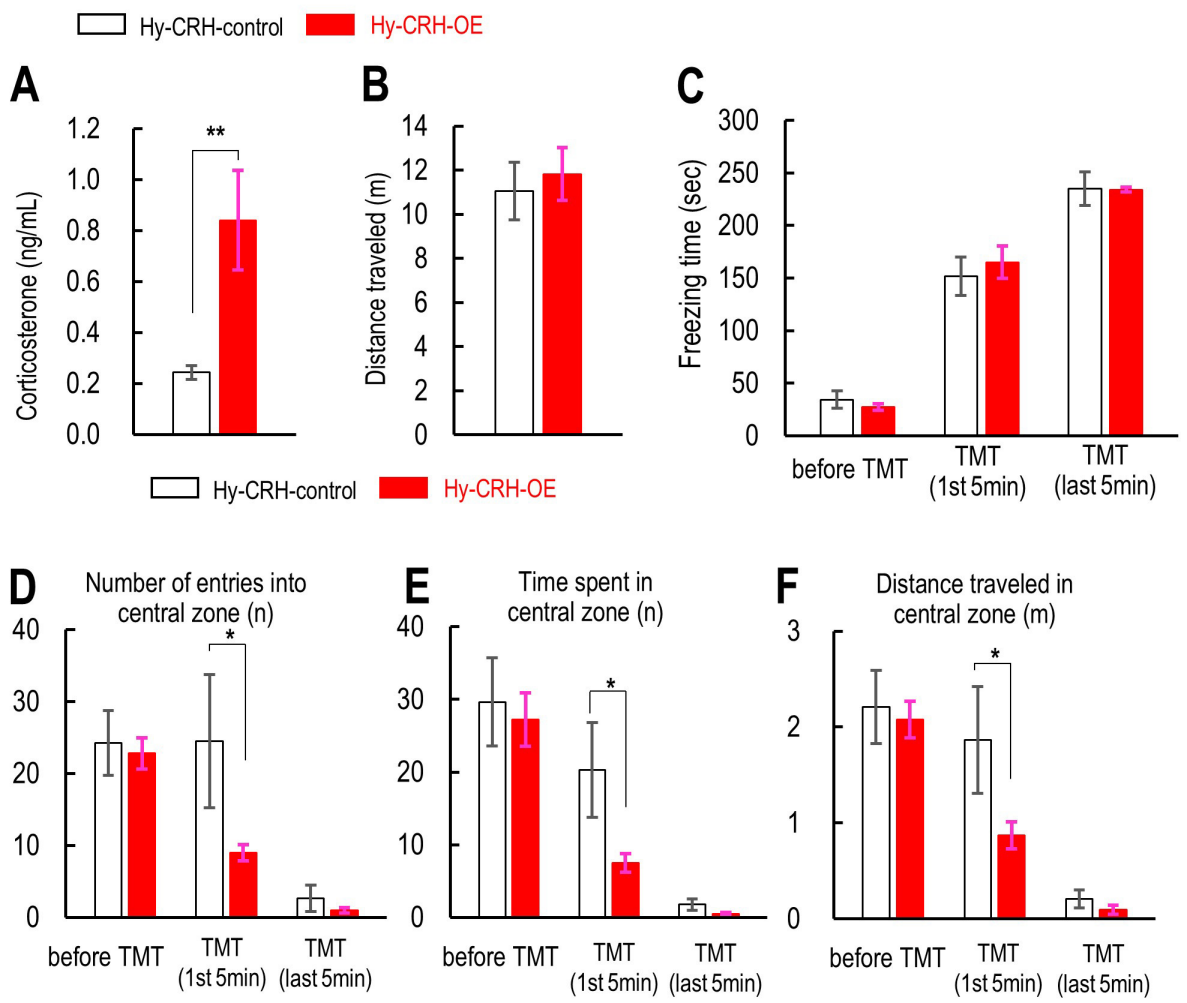
**The effect of hypothalamic CRH on locomotor activity, fear response levels, and central preference**. (A) Concentration of plasma corticosterone in control virus injection into the hypothalamus (Hy-CRH-control) (n = 5) and the overexpression of corticotropin-releasing hormone in hypothalamus (Hy-CRH-OE) (n = 5) mice during 12:00–14:00 h in the light phase. (B) Effect of hypothalamic CRH overexpression on locomotor activity. Distance (m) traveled in an open field test box. Hy-CRH-control (n = 8) and Hy-CRH-OE (n = 10). (C) The percentage of freezing time for 5 min before (–5 to 0 min) and during the first (1‒5 min) and last 5 min (6–10 min) of mineral oil (MO) and 2,5-dihydro-2,4,5-trimethylthiazoline (TMT) exposure in Hy-CRH-control mice (MO: n = 6; TMT: n = 8) and Hy-CRH-OE mice (MO: n = 7; TMT: n = 10). (D–F) Distance traveled (D), number of entries (E), and time spent (F) in the central zone in the open field test box for 5 min before (‒5 to 0 min) and during the first and second 5 min of MO and TMT exposure in Hy-CRH-control (MO: n = 6; TMT: n = 8) and Hy-CRH-OE mice (MO: n = 7; TMT: n = 10). Data are presented as mean ± SEM, with statistical significance indicated (*p < 0.05, **p < 0.01). CRH, corticotropin-releasing hormone; TMT, 2,5-dihydro-2,4,5-trimethylthiazoline; MO, mineral oil.

Psychological stress induces corticotropin-releasing hormone (CRH) release in the paraventricular nucleus of the hypothalamus, leading to increased corticosterone levels in mice. In our previous study, we generated the adeno-associated virus (AAV) vector for CRH overexpression and injected it into the hypothalamus (**Supplementary Fig. 1**) [24]. Therefore, to assess baseline corticosterone levels, we used the mice with either the control virus injection into the hypothalamus (Hy-CRH-control) or hypothalamic CRH overexpression (Hy-CRH-OE) under non-stressful conditions. Hy-CRH-OE mice exhibited a significantly higher plasma corticosterone concentration than Hy-CRH-control mice (*t*_(8)_ = 46.6, *p* < 0.05, Cohen’s *d* = 2.14, minimum sample size per group calculated by a priori sample size; n = 5, Student *t*-test: *p* < 0.01, Fig. 1A). However, Hy-CRH-OE did not affect the locomotor activity, as measured by the distance traveled in the OF test (*t*_(16)_ = 46.6, *p* = 0.8, Cohen’s *d* = 0.011, Student *t*-test: *p* = 0.9769, Fig. 1B). Three-way repeated measures ANOVA (Odor [mineral oil or TMT] × Genotype × Time) revealed that the interaction between odor and time significantly affected fear sensitivity (*F*_(3,92)_ = 46.5, *p* < 0.01, Fig. 1C). However, a two-way ANOVA (Odor × Genotype) for period of “before odor” indicated that freezing time was not significantly affected (*F*_(3,68)_ = 0.422, *p* = 0.738, Fig. 1C). In contrast, during both the initial (1–5 min) and final (6–10 min) period, freezing time was significantly affected (1–5 min: *F*_(3,68)_ = 33.85, *p* < 0.01; 6–10 min: *F*_(3,68)_ = 76.16, *p* < 0.01; Fig. 1C). A Bonferroni post-hoc test indicated that TMT significantly increased freezing time in both the initial and final phases (1–5 min: Hy-CRH-control TMT, *p* < 0.01 vs. Hy-CRH-control MO, *p* < 0.01 vs. Hy-CRH-OE MO; Hy-CRH-OE TMT, *p* < 0.01 vs. Hy-CRH-OE MO; Fig. 1C). Similarly, three-way repeated measures ANOVA (Odor [mineral oil or TMT] × Genotype × Time) showed significant effects of odor on central preference measures: number of entries (*F*_(3,92)_ = 7.26, *p* < 0.01, Fig. 1D), time spent (*F*_(3,92)_ = 14.95, *p* < 0.01, Fig. 1E), and distance traveled (*F*_(3,92)_ = 5.94, *p* < 0.01, Fig. 1F). Two-way ANOVA for the pre-odorant phase revealed no significant effects on central preference (number of entries: *F*_(3,70)_ = 0.055, *p* = 0.983; time spent: *F*_(3,78)_ = 0.085, *p* = 0.968; distance traveled: *F*_(3,61)_ = 0.043, *p* = 0.988; Fig. 1D–F). However, significant effects were observed during both the initial (1–5 min) and final (6–10 min) period (1–5 min: number of entries: *F*_(3,70)_ = 5.86, *p* < 0.01; time spent: *F*_(3,78)_ = 9.95, *p* < 0.01; distance traveled: *F*_(3,61)_ = 4.68, *p* < 0.01; 6–10 min: number of entries: *F*_(3,70)_ = 15.35, *p* < 0.01; time spent: *F*_(3,78)_ = 20.19, *p* < 0.01; distance traveled: *F*_(3,61)_ = 14.62, *p* < 0.01; Fig. 1D–F). Bonferroni post-hoc tests revealed that TMT significantly reduced central preference compared to mineral oil in both periods (1–5 min: Hy-CRH-control TMT, *p* = 1.000 vs. Hy-CRH-OE MO; Hy-CRH-OE TMT, *p* < 0.01 vs. Hy-CRH-control MO, *p* < 0.01 vs. Hy-CRH-OE MO; 6–10 min: Hy-CRH-control TMT, *p* < 0.01 vs. Hy-CRH-control MO, *p* < 0.01 vs. Hy-CRH-OE MO; Hy-CRH-OE TMT, *p* < 0.01 vs. Hy-CRH-control MO, *p* < 0.01 vs. Hy-CRH-OE MO; Fig. 1D–F).

### 3.2 Hypothalamic CRH Overexpression Facilitates Passive Coping During Aversive Stress After TMT-Evoked Fear Experience

**Fig. 2.**
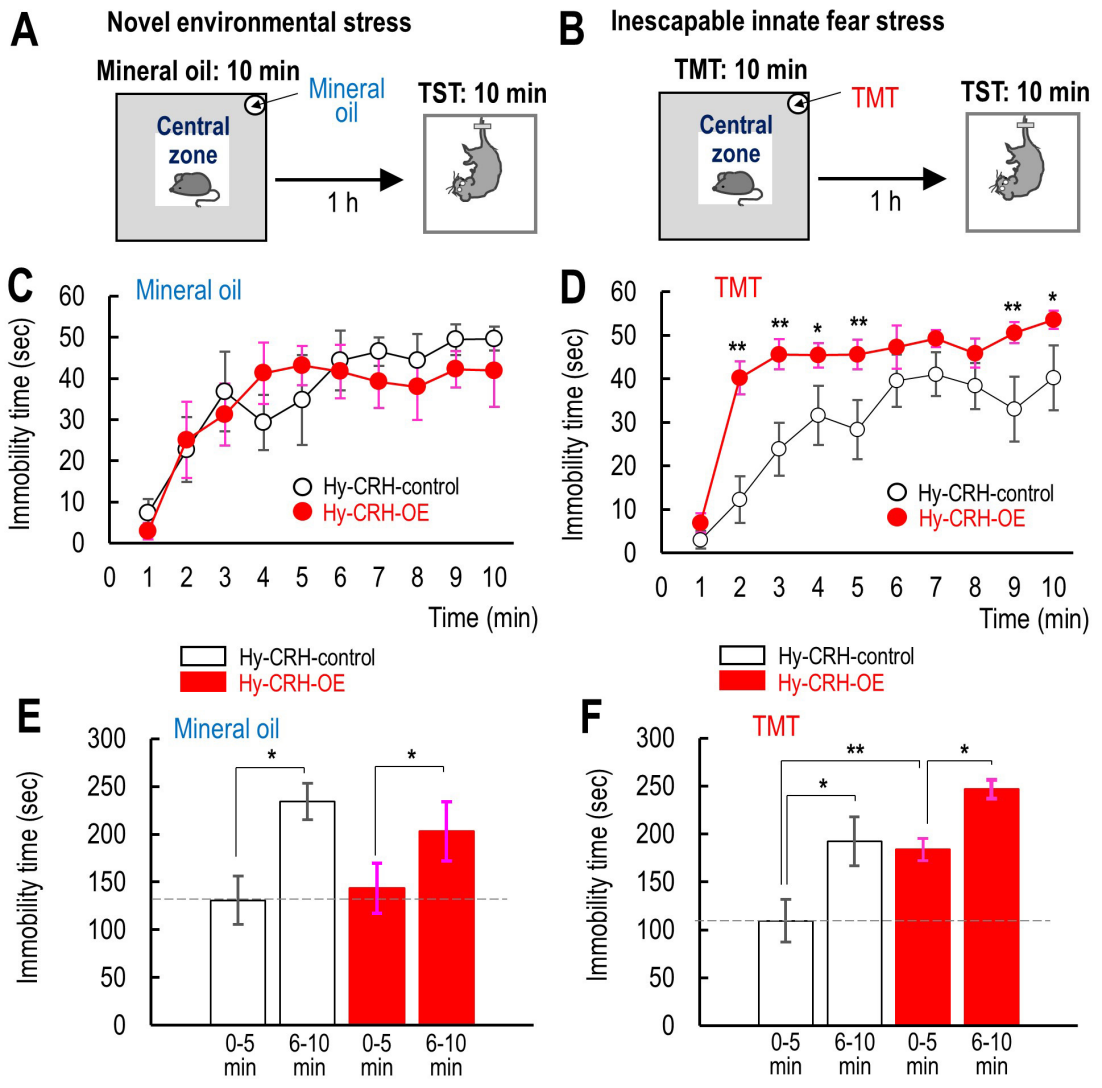
**The effect of CRH overexpression in the hypothalamus on active coping levels during the TST after TMT-evoked fear stress**. (A,B) Schematic diagrams indicate the placement of the central zone and either MO (A) or TMT (B), as well as the schedule for the tail suspension test (TST). (C,D) Demonstrate time-dependent changes in immobility over the 10-minute period of the TST in Hy-CRH-control (n = 7) and Hy-CRH-OE mice (n = 6) under the presence of MO (C), and in Hy-CRH-control (n = 8) and Hy-CRH-OE mice (n = 10) under TMT exposure (D). (E,F) Overview of immobility duration during the first (1–5 min) and final 5 minutes (6–10 min) 1 hour after exposure to TMT in Hy-CRH-control (n = 7) and Hy-CRH-OE mice (n = 6) under the presence of MO (E), and in Hy-CRH-control (n = 8) and Hy-CRH-OE mice (n = 10) under TMT exposure (F). Data are presented as mean ± SEM, with statistical significance denoted (*p < 0.05, **p < 0.01).

Moderate fear experiences have been shown to decrease immobility time in rats [25], whereas it has been demonstrated that more severe fear experiences result in increased time spent immobile during the forced swimming test (FST), accompanied by elevated corticosterone levels, which affect coping behavior under subsequent inescapable stress [26]. Thus, corticosterone levels may influence passive coping levels during the TST following TMT-evoked fear experience. We measured immobility time during TST following exposure to the OF test (Fig. 2A) (mineral oil as a control of TMT exposure (Fig. 2B)) in Hy-CRH-control and Hy-CRH-OE mice. When the TMT was not introduced, no significant differences in immobility time were observed between the two groups (Mineral oil: two-way repeated measures ANOVA: *F*_(1,129)_ = 0.0722, *p* = 0.793, Fig. 2C), suggesting that Hy-CRH-OE did not affect passive coping during aversive stress. Coping level in the last 5 min of TMT exposure (6–10 min) was similar between groups when the TMT was not present (Mineral oil: *F*_(1,25)_ = 0.073, *p* = 0.792, Fig. 2E), indicating that the learned despair level was comparable between both groups. This suggests that the increase in passive coping behavior during the final 5 minutes reflects learned despair [19]. However, after TMT exposure, which evoked inescapable innate fear, Hy-CRH-OE mice exhibited a significantly longer immobility time during TST compared to Hy-CRH-control mice at all time points (two-way repeated measures ANOVA: *F*_(1,179)_ = 6.80, *p* < 0.05, Fig. 2D). Post hoc tests by student *t*-test revealed significant differences at multiple time points, with Hy-CRH-OE mice showing longer immobility time than controls (TMT: *p* < 0.01 for 2, 3, 5, 9 min and *p* < 0.05 for 10 min, Fig. 2D). Furthermore, during the initial 5 min of TST (1–5 min), Hy-CRH-OE mice also displayed significantly higher immobility time compared to Hy-CRH-control mice after TMT exposure (TMT: two-way repeated measures ANOVA: *F*_(1,35)_ = 6.81, *p* < 0.05; Bonferroni post hoc test: 1–5 min: TMT: *p* < 0.01 vs. control, Fig. 2F). Taken together, since the increased immobility time in Hy-CRH-OE mice was observed both in the first and last 5 minutes, with significantly longer times compared to Hy-CRH-Control mice, prior exposure to TMT-induced fear stress reduced active coping abilities in the Hy-CRH-OE mice during the subsequent aversive stress in the TST, rather than enhancing the learned despair level.

### 3.3 Correlation Between Freezing Time, Central Preferences During Fear Stress, and Passive Coping Behavior During Subsequent Aversive Stress

**Fig. 3.**
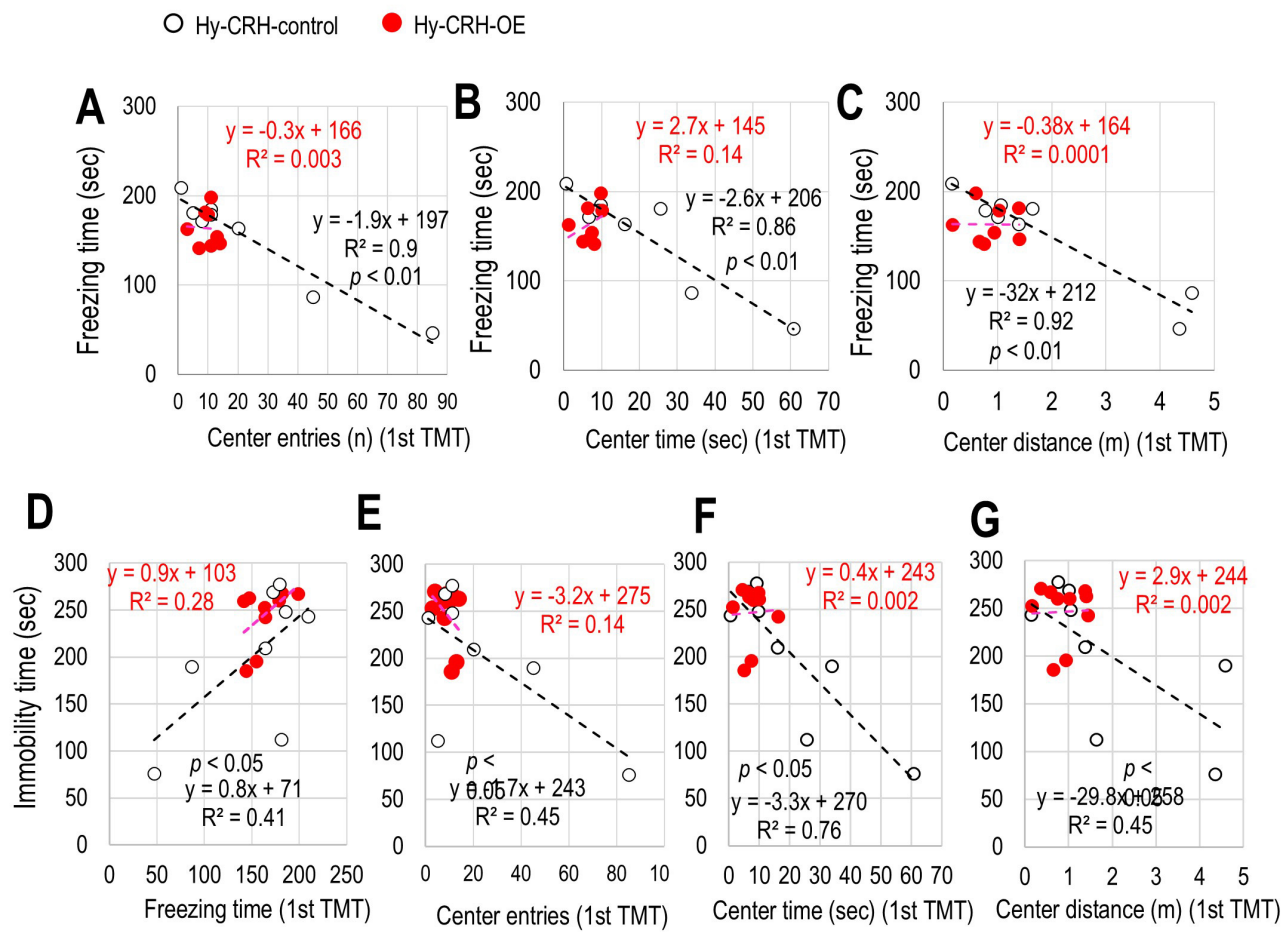
**The effect of CRH overexpression in the hypothalamus on the relationships between freezing levels, central preference, and active coping levels**. (A–C) Correlation between the percentage of freezing time in the first 5 minutes of TMT exposure and the number of entries (A), the time spent (B), and the distance traveled (C) in the central zone in the open field test box for both Hy-CRH-control (n = 8) and Hy-CRH-OE (n = 10) mice. (D) Correlation between the percentage of freezing time and immobility duration during the initial 5 minutes of TMT exposure for both Hy-CRH-control (n = 8) and Hy-CRH-OE mice (n = 10). (E–G) Correlation between the number of entries (E), the time spent (F), and the distance traveled (G) in the central zone during the first 5 min of TMT exposure and the immobility time during the first 5 min of the tail suspension test (TST) in Hy-CRH-control (n = 8) and Hy-CRH-OE mice (n = 10). R^2^ is Pearson’s correlation coefficient.

To further examine the association between fear responses and coping behavior, we examined whether there were correlations between central preferences during TMT-evoked inescapable innate fear stress and passive coping behavior during the subsequent TST. In Hy-CRH-control mice, significant correlations were observed between central preferences (number of entries, time spent, and distance traveled in the central zone) and freezing time during TMT exposure (Hy-CRH-Control: Shapiro-Wilk test: freezing time: *p* < 0.05, number of entries: *p* < 0.05, time spent: *p* < 0.05, distance traveled: *p* < 0.05; Pearson’s correlation coefficient: number of entries: *p* < 0.01, Fig. 3A; time spent: *p* < 0.01, Fig. 3B; distance traveled: *p* < 0.01, Fig. 3C, Table 1), indicating that a high level of central preference was associated with a relatively lower freezing time. Moreover, there was a correlation between immobility time in the TST and freezing time during TMT exposure (Hy-CRH-Control: Shapiro-Wilk test: immobility time: *p* < 0.05; Pearson’s correlation coefficient: number of entries: *p* < 0.01; Fig. 3D) as well as with central preferences (Pearson’s correlation coefficient: number of entries: *p* < 0.05, Fig. 3E; time spent: *p* < 0.05, Fig. 3F; distance traveled: *p* < 0.05, Fig. 3G, Table 1). However, the correlations between freezing time and central preference, as well as between immobility time and either freezing time or central preference, which were observed in the Hy-CRH-Control mice, were completely abolished in the Hy-CRH-OE mice (Fig. 3A–G, Table 1). These results suggest that low corticosterone concentrations in Hy-CRH-control mice during TMT-induced fear stress or high corticosterone levels in Hy-CRH-OE mice without fear stress (mineral oil instead of TMT) may result in similar correlations between central preference and passive coping levels during the TST. However, sustained high corticosterone levels following TMT exposure seem to disrupt these correlations in Hy-CRH-OE mice.

**Table 1.** **Pearson’s correlation coefficient between behavior1 and 2 in Hy-CRH-Control and Hy-CRH-OE mice**.

Group	TMT or TST	TMT	R^2^	*p*
Hy-CRH-Control	TMT	No. entries in central zone	0.946	< 0.001**
		Time spent in central zone	0.893	< 0.001**
		Distance traveled in central zone	0.933	< 0.001**
	TST	Freezing time	0.579	< 0.05*
		No. entries in central zone	0.565	< 0.05*
		Time spent in central zone	0.763	< 0.01**
		Distance traveled in central zone	0.552	< 0.05*
Hy-CRH-OE	TMT	No. entries in central zone	0.022	0.69
		Time spent in central zone	0.028	0.65
		Distance traveled in central zone	0.008	0.81
	TST	Freezing time	0.279	0.12
		No. entries in central zone	0.138	0.29
		Time spent in central zone	0.002	0.90
		Distance traveled in central zone	0.002	0.91

TST, tail suspension test. R^2^, coefficient of determination. *p* < 0.05*, < 0.01**.

### 3.4 The Role of MR and GR in the Correlation Between Central Preference and Freezing Time During TMT-Induced Innate Fear Stress

**Fig. 4.**
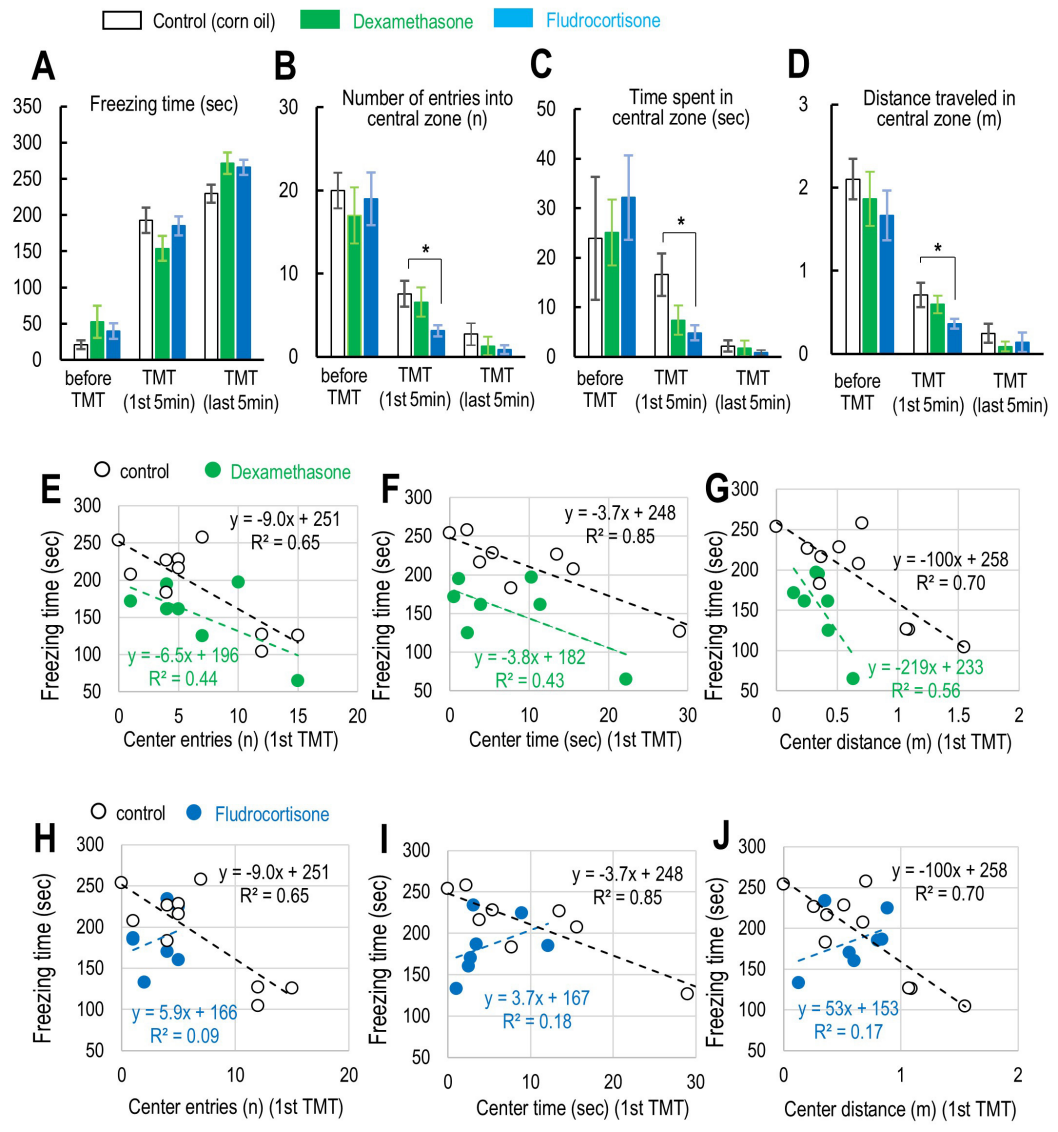
**The contribution of glucocorticoid or mineralocorticoid receptors to the correlation between freezing levels and central preferences during TMT exposure**. (A–D) Freezing time (sec) (A), number of entries (B), time spent (C), and distance traveled (m) (D), in the central zone in the open field test box before (‒5 to 0 min) and during the first and second 5 min of TMT exposure in control (n = 10), dexamethasone-treated (n = 7), and fludrocortisone-treated (n = 7) mice. (E–G) Correlation between the number of entries (E), the time spent (F), and the distance traveled (G) in the central zone during the first 5 min of TMT and the freezing time during the first 5 min of TMT in control (n = 10) and dexamethasone-treated mice (n = 7). (H–J) Correlation between the number of entries (H), the time spent (I), and the distance traveled (J) in the central zone and immobility time during the first 5 min of the TST in control (n = 10) and fludrocortisone-treated mice (n = 7). Data are presented as mean ± SEM, with statistical significance indicated (*p < 0.05).

The abolition of the correlation of central preference during TMT-evoked innate fear stress and passive coping behavior during subsequent aversive stress in the TST was due to hypothalamic CRH overexpression. To identify the corticoid receptor contributing to these correlations in Hy-CRH-OE mice, we administered subcutaneous injections of dexamethasone (a GR agonist, 10 µg/kg) or fludrocortisone (an MR agonist, 5.0 mg/kg) to non-AAV-infected normal mice 30 minutes before TMT-induced innate fear stress. This was based on evidence that 10 µg/kg of dexamethasone is the minimum dose that does not affect active coping levels in naïve mice without prior psychological stress [10], while 5.0 mg/kg of fludrocortisone is the minimum dose required to induce renal effects in mice [27]. Given that central preference in Hy-CRH-OE mice was lower than in Hy-CRH-control mice, we investigated which receptor mediates the reduction in central preference during TMT-evoked inescapable innate fear stress. Two-way repeated measures ANOVA revealed that the interaction between time and drug did not affect fear sensitivity (*F*_(2,71)_ = 1.23, *p* = 0.3113, Fig. 4A). Furthermore, two-way repeated measures ANOVA indicated that the interaction between time and drug did not affect the number of entries into the central zone (*F*_(2,71)_ = 2.081, *p* = 0.150, Fig. 4B). However, one-way ANOVA revealed significant effects of drug on the central preference during first 5 min of TMT exposure, including the number of entries into the central zone (*F*_(2,23)_ = 9.311, *p* < 0.01, Fig. 4B), time spent in the central zone (*F*_(2,23)_ = 4.94, *p* < 0.05, Fig. 4C), and distance traveled in the central zone (*F*_(2,23)_ = 4.39, *p* < 0.05, Fig. 4D). Bonferroni post hoc tests indicated that fludrocortisone-treated mice exhibited reduced central preference, as evidenced by fewer entries (*p* < 0.05), less time spent (*p* < 0.05), and shorter distance traveled (*p* < 0.05) in the central zone, compared to controls (Fig. 4B‒D). In contrast, dexamethasone-treated mice showed no significant differences compared to controls in the central preferences (Fig. 4B‒D). Furthermore, the significant correlations between central preference and freezing time during TMT-evoked inescapable innate fear stress were observed in dexamethasone-treated mice as well as control mice (Fig. 4E‒G and Table 2). On the other hand, pretreatment of fludrocortisone abolished these correlations between central preference and freezing time during TMT-evoked inescapable innate fear stress (Fig. 4H‒J and Table 2). These findings suggest that disrupted correlations between freezing time and central preference in Hy-CRH-OE mice may be due to an imbalance between MR and GR activity, potentially resulting in MR hyperactivation during TMT-induced inescapable innate fear stress.

**Table 2.** **Pearson’s correlation coefficient between behavior 1 and 2 in control, dexamethasone or fludrocortisone treated mice**.

Group	TMT or TST	TMT	R^2^	*p*
Control	TMT	No. entries in central zone	0.534	< 0.05*
		Time spent in central zone	0.847	< 0.01**
		Distance traveled in central zone	0.698	< 0.01**
	TST	Freezing time	0.784	< 0.01**
		No. entries in central zone	0.780	< 0.01**
		Time spent in central zone	0.716	< 0.01**
		Distance traveled in central zone	0.608	< 0.01**
Dexamethasone	TMT	No. entries in central zone	0.801	< 0.01**
		Time spent in central zone	0.568	< 0.05*
		Distance traveled in central zone	0.559	< 0.05*
	TST	Freezing time	0.609	< 0.05*
		No. entries in central zone	0.637	< 0.05*
		Time spent in central zone	0.601	< 0.05*
		Distance traveled in central zone	0.730	< 0.05*
Fludrocortisone	TMT	No. entries in central zone	0.097	0.496
		Time spent in central zone	0.178	0.346
		Distance traveled in central zone	0.174	0.352
	TST	Freezing time	0.330	0.178
		No. entries in central zone	0.098	0.495
		Time spent in central zone	0.431	0.109
		Distance traveled in central zone	0.074	0.554

*p* < 0.05*, < 0.01**.

### 3.5 The Role of MR and GR in the Correlation Between Central Preference Under Fear Stress and Passive Coping Behaviors in Subsequent Aversive Stress Exposure

**Fig. 5.**
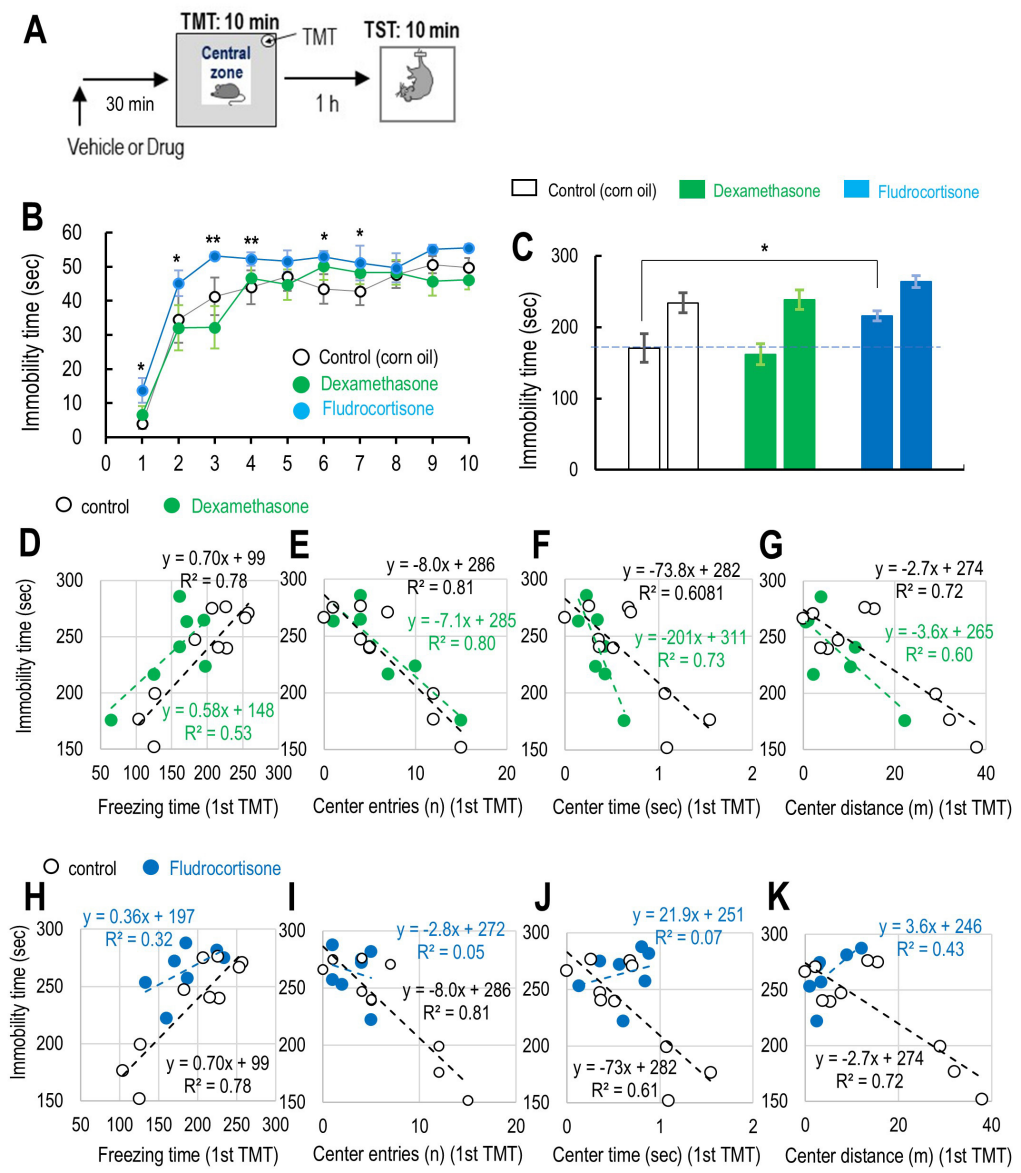
**The contribution of glucocorticoid or mineralocorticoid receptors to the relationship between freezing levels, central preferences during TMT exposure, and active coping levels during the TST**. (A) Schematic drawings indicate the schedule of vehicle or drugs, 5 min of open field test before TMT exposure, followed by 10 min of TMT exposure and the tail suspension test (TST). (B) The time-dependent changes in immobility during the 10-minute TST in the control group (n = 10), dexamethasone-treated (n = 7), and fludrocortisone-treated (n = 7) mice under TMT exposure. (C) Summary of immobility time during the initial (1–5 min) and final (6–10 min) 5 minutes, 1 hour after TMT exposure in control (n = 10), dexamethasone-treated (n = 7), and fludrocortisone-treated (n = 7) mice. (D‒K) Correlation between freezing time during the first 5 min and the immobility time during the first 5 min of TMT exposure for control (n = 10), dexamethasone-treated (n = 7) (D‒G), and fludrocortisone-treated (n = 7) mice (H‒K). Correlation between the freezing time (D,H), the number of entries (E,I), the time spent (F,J), and the distance traveled (G,K) in the central zone during the first 5 min of TMT exposure and the immobility time during the first 5 min of the TST in control and fludrocortisone-treated mice. Data are presented as mean ± SEM, with statistical significance indicated (*p < 0.05, **p < 0.01).

Next, we investigated the roles of MR and GR in passive coping responses following fear-induced stress and examined the correlation between central preference and passive coping levels (Fig. 5A). A two-way repeated measures ANOVA revealed no significant interaction between time and drug on immobility time in the TST (*F*_(2,239)_ = 1.764, *p* = 0.196, Fig. 5B). However, Bonferroni post hoc tests indicated that fludrocortisone significantly increased immobility time at multiple time points compared to the control group (1 min: *p* < 0.05; 2 min: *p* < 0.05; 3 min: *p* < 0.01; 4 min: *p* < 0.01; 6 min: *p* < 0.05; 7 min: *p* < 0.05; Fig. 5B). Fludrocortisone also reduced active coping level, as evidenced by increased immobility time during the TST (*F*_(2,71)_ = 2.158, *p* = 0.141, Bonferroni post hoc test: *p* < 0.05 vs. control, Fig. 5C). The correlation between immobility time in the TST and freezing time during TMT-evoked fear stress remained unaffected by dexamethasone (Fig. 5D, Table 2), as did the correlation between immobility time in the TST and central preference during TMT-induced fear stress (Fig. 5E‒G, Table 2). However, pretreatment with fludrocortisone abolished both the correlation between immobility time in the TST and freezing time during TMT-evoked fear stress (Fig. 5H, Table 2) and the correlation between immobility time and central preference during TMT-evoked fear stress (Fig. 5I‒K, Table 2). These findings suggest that MR activation disrupts the relationship between central preference during inescapable innate fear stress and passive coping during subsequent aversive stress.

## 4. Discussion

In the present study, investigating the relationship between mouse behavior during acute fear stress and subsequent stress-coping levels under aversive conditions may provide insights into how our experimental model of mouse behavior during acute psychological stress influences the promotion of active coping or the prevention of passive coping during subsequent psychological stress. While repeated and chronic psychological stress is considered a contributing factor in the development of MDD [28], it is possible that if individuals can adapt to acute psychological stress by engaging in appropriate coping behaviors, it might be a means of preventing the onset of MDD. The ability to cope with stress-induced helplessness is associated with an active coping style [4], and active coping behaviors are associated with increased resilience to stress [1]. Active coping represents a behavioral response that mitigates the physical, psychological, or social detriment of a situation [1]. In the present study, we have shown that the higher central preferences in the OF-test box during preceding psychological fear stress exhibited lower passive coping levels, meaning higher active coping in the subsequent TST as aversive stress. These results suggest that the extent of central area exploration during TMT-evoked fear stress is a key factor in reducing passive coping behaviors during subsequent stress.

It has been demonstrated that acute and chronic stress can differently affect immobility time during the TST in rats [25]. In mice, exposure to acute footshock or acute restraint immediately before the test significantly reduces immobility in the TST [25]. Even when mice have prior experience with chronically repeated footshocks, acute footshock still results in a shortened duration of immobility in the TST [25]. Interestingly, both chronic treatment with metyrapone, a glucocorticoid synthesis inhibitor, for 14 days, and acute treatment with metyrapone combined with corticosterone significantly reduce immobility time during the FST in rats [29]. Consistently, animals subjected to chronic corticosterone treatment exhibited prolonged immobility time during the FST [30]. It has also been demonstrated that intracerebroventricular injection of CRH decreased the immobility time during the TST [25]. This occurs because a moderate level of electrical shock results in a shorter immobility duration observed in untreated male rats. It has been shown that intense fear induced by electrical shocks contributes to increased immobility time during the FST. This effect is accompanied by a rise in plasma corticosterone levels, which, in turn, influences coping behavior in response to subsequent inescapable stress [26]. Thus, varying doses of metyrapone or CRH may produce different outcomes. As numerous studies have shown, prolonged psychological stress is one of the pathophysiological mechanisms of MDD [28, 29]. The increased immobility time during the TST or FST in rodents after chronic psychological stress is often interpreted as a manifestation of behavioral despair. These tests serve as animal models for MDD [31, 32, 33]. In the present study, acute TMT-evoked fear stress did not have a significant impact on the duration of immobility time in the TST when compared to mice that were not exposed to TMT. This suggests that TMT-evoked fear stress may not be severe or moderate for mice, and TMT exposure might constitute a relatively weak stressor.

This correlation was abolished in mice with overexpression of hypothalamic CRH and fludrocortisone-treated mice. These results indicate that the higher activation of MR rather than GR during psychological fear stress is harmful to induce the passive coping behavior. Elevated cortisol levels have also been proposed to contribute to the psychosis seen in psychotic MDD [34]. In non-stress conditions, corticosteroids have a higher affinity for MR than GR at low plasma levels, but after the stress, GR becomes more occupied by corticoid hormone [35], indicating that the acute stress changes the balance between MR/GR activities to adapt to the stress. Therefore, if this balance of MR/GR might be not altered in the Hy-CRH-OE mice, it suggests that the homeostasis of the HPA axis is not naturally adapted to cope with stress, which could be detrimental to promoting desirable behavioral responses to stress. It has been suggested that GR activation promotes memory consolidation and behavioral adaptation [36], and a few recent reports using pharmacological agents to block receptor activity suggest that MR may be involved in the passive coping response [37].

Most studies have consistently demonstrated that GR function is compromised in major depression, with reduced GR-mediated feedback in the HPA axis [36]. According to several studies, GR agonists induce acute antidepressant effects. Dexamethasone, a synthetic glucocorticoid, has been administered in combination with sertraline and fluoxetine for four days [38] or without dexamethasone treatment, a single intravenous injection of cortisol rapidly enhances depressive symptom improvement in patients with depression [39]. As a pretreatment approach, stress inoculation improves the ability to cope with stress and regulate emotions in subsequent situations [40]. A lot of studies have confirmed the role of GR in depression. However, in recent years, it has been suggested that the dysfunctional activity of MR contributes substantially to the pathophysiology of depression [41]. Preclinical and clinical studies have suggested a role of MR activation in the response to antidepressants [42]. In contrast, administering spironolactone, an MR antagonist, also confers an antidepressant effect [30]. However, how the MR contributes to stress-induced mood disorder remains poorly understood. Acute stress has been shown to elevate MR expression levels in the rat hippocampus, subsequently enhancing in the inhibitory influence exerted by MR on HPA axis activity [43]. Increased activation of postsynaptic serotonin (5-HT) 1A receptor (5-HT1AR) is thought to contribute to a tendency toward an anxious phenotype and reduced ability to cope with stress [44]. Prolonged exposure to elevated corticosterone levels attenuates 5-HT1A responses [45]. Although decreased brain derived neurotrophic factor (BDNF) levels are believed to be related to mood disorders and symptom severity [46], increased MR expression and a trend toward increased BDNF expression [47]. The anxiogenic effects of BDNF observed in both tests were attenuated by pre-administration of the 5-HT1A receptor antagonist [48]. Therefore, MR activation-induced abnormalities in both 5-HT1A and BDNF may serve as potential targets for acute stress adaptation to prevent MDD in future studies.

According to recent findings, it has been proposed that the rodent forced swimming test measures the stress-coping strategies rather than depression-like behavior [49]. The behavioral changes assessed by the FST may thus be interpreted as reflecting the adaptability of stress-coping mechanisms [50]. The TST follows a similar conceptual framework in rodent studies. Therefore, our observations from the TST experiments suggest that the observed behaviors reflect coping strategies rather than depressive-like states. Therefore, our observations from the TST experiments indicate coping behavior rather than depressive-like behavior. Because the Hy-CRH-control mice exhibited a negative correlation between central preference during acute psychological fear stress and passive coping in subsequent aversive stress, higher activity of GR, rather than MR, appears to be more critical for adapting to acute stress. However, while we identified an association between higher central preferences and strong active coping behavior, it would be advantageous in future studies to predict the dose-dependent effects of GR activity on central preferences during preceding acute fear stress.

Sex differences in response to fear-related stress have been suggested [15]. Furthermore, when rats were exposed to chronic unpredictable stress during early life, females exhibited MR downregulation, whereas males showed MR upregulation as they matured [51]. Women also display differences in HPA axis regulation based on menopausal status, with menopausal women being more likely to be corticotropin non-suppressors compared to pre-menopausal women [52]. Additionally, women in the luteal phase exhibit decreased cortisol suppression in the DST compared to the follicular phase [53]. GR has been shown to alter estrogen gene expression [54]. Therefore, investigating sex differences in stress adaptation and coping through corticosteroid receptors is essential. However, because investigating the role of corticosteroids in acute stress adaptation and coping is more complex in female mice than in male mice, the present study focused on the effects of stress hormones. To simplify the investigation of the role of stress hormones while minimizing complexities and variations in stress responses across different phases of the estrous cycle and accounting for the influence of sex hormones, we exclusively used male mice in our experiments.

## 5. Conclusions

Here we identified a pivotal association between behavioral patterns exhibited during acute psychological fear stress and subsequent levels of active coping under aversive stress. Our findings underscore how reactions to acute psychological stress can promote active coping or hinder the formation of passive coping strategies during later aversive stressors. We also demonstrated that HPA axis activation disturbs the connection between behavioral patterns during prior psychological fear stress and active coping levels during subsequent aversive stress through MR.

## Availability of Data and Materials

Data are available from the corresponding author upon a reasonable request and with the permission of Tohru Matsuki and Kenjiro Seki.

## Supplementary Material

Supplementary material associated with this article can be found, in the online version, at https://doi.org/10.31083/AP46061.
