# Supplementary material for: Adaptation to Preceding Acute Psychological Stress is Associated With Subsequent Stress Coping Levels via Corticoid Receptors
Source: Alpha Psychiatry. 2025 Aug 26;26(4):46061. doi: 10.31083/AP46061 (PMC12416057; doi:10.31083/AP46061)
Supplement: Supplementary file 1 [file 2757-8038-26-4-46061-s1.zip › Supplementary material.docx]

**Title:** **Adaptation to preceding acute psychological stress is associated with subsequent stress coping levels via corticoid receptors**

**Supplementary Materials**

**Methods and Materials**

**Producing the Adeno-associated virus (AAV) for Overexpression of CRH**

All recombinant DNA experiments were performed in accordance with the guidelines of the Recombinant DNA Experiments Committee of Ohu University (No. 2021004) and Aichi Developmental Disability Center (No. 19-6), in compliance with the criteria mandated by the Act on the Conservation and Sustainable Use of Biological Diversity through Regulations on the Use of Living Modified Organisms in Japan. We have already reported about the plasmid for inducing the overexpression of CRH using the Adeno-associated virus (AAV) ^1^. In brief, the plasmid to express CRH-T2A-RFP (red fluorescent protein) was developed by modifying a plasmids pUCmini-iCAP-PHP.eB (Addgene #103005, Watertown, MA, USA). We substituted the RNA polymerase III-driven U6 promoter encoding the synthesized DNA fragment encoding the U6 promoter, CAG promoter, CRH-T2A-RFP (Biomatik, Cambridge, ON, Canada). We previously demonstrated that the CRH expression was analyzed by immunoblot with anti-FLAG-HRP antibody ^1^. AAV was produced by referring the paper by Challis et al.^2^. HEK293FT cells were transfected with the plasmids pUCmini-iCAP-PHP.eB, pAdDeltaF6 (#112867, Addgene), and the plasmid-carrying rAAV genome (pAAV control). Cell pellets were dissociated with 1 mg/mL of DNase I solution and subjected to six freeze–thaw cycles. The AAV-containing solution was collected and placed at 4 °C. The culture medium was mixed with 40% PEG8000, followed by incubation on ice for 2 h. AAV particles were precipitated by centrifugation at 6000× g, 4 °C for 30 min with DNase I solution. The AAV-containing solution were ultracentrifuged at 160,000× g with Opti Prep gradients. Purified AAV was concentrated and buffer-exchanged to PBS (−). The virus was titrated using THUNDERBIRD SYBR qPCR Mix (Toyobo, Osaka, Japan) and a CFX96 real-time PCR system (Bio-Rad Laboratories, Hercules, CA, USA), with the following primer sets targeting the WPRE sequence: GGCTGTTGGGCACTGACAAT and CCGAAGGGACGTAGCAGAAG.

**AAV Injection into the Hypothalamus**

In accordance with the guidelines of the Animal Care Committee of Ohu University, as we previously reported ^1^, mice were anesthetized with a mixture of medetomidine hydrochloride, butorphanol tartrate (0.3 and 5.0 mg/kg, respectively; Wako Pure Chemical Corp.) and midazolam (4.0 mg/kg; Sandoz Ltd., Yamagata, Japan) to ensure the loss of sensation, including loss of pain sensation and immobilization during procedures. After anesthetization, the mice were placed in a stereotactic frame (#68045, RWD Life Science, Guangdong, China), A Hamilton neurosyringe (32-gauge, 7000.5 Neuros Syringe, #65457-02, Hamilton Co. Japan K.K., Tokyo, Japan) was used for bilateral AAV microinjection into the bilateral hypothalamus, which was carried out by infusing 0.1 μL AAV into each side, which was taking 5 min for 0.1 μL injection of AAV solution into hypothalamus. The titers of AAV-Control-GFP were 8.32 × 10^12^ vg/mL and of AAV-CRH-RFP (for overexpression of CRH) were 3.49 × 10^12^ vg/mL and respectively. The stereotaxic coordinates (mm) for hypothalamic injection, according to the Paxinos mouse brain atlas ^3,4^, were anteroposterior (AP), −0.7 mm, lateral (L), 0.25 mm from bregma; depth and (DV), −4.6 mm. After the AAV injection, the neurosyringe was left in place for at least 5 min to minimize the spread and leakage of the drug along the injection track ^5^. Next, both holes were filled with Dental cement (GC Unifast II, GC Dental Products Corp., Tokyo, Japan) were used for filled the hole for injection and fixed by the stabilizing screw. After waking from anesthesia, mice were kept in their home cages for at least 14 days before behavioral tests.

**Verification of AAV Infection and CRH Expression**

We performed the immunofluorescence staining by the standard protocol as we previously reported ^1^. In brief, mice were anesthetized with a mixture of medetomidine hydrochloride, butorphanol tartrate (0.3 and 5.0 mg/kg, respectively), and midazolam (4.0 mg/kg). Transcardial perfusion was performed with ice-cold 0.1 M PBS, followed by 4% ice-cold PFA in 0.1 M PBS. The brains were removed and post-fixed in OTC compound (Sakura Finetek Japan Co., Ltd., Tokyo, Japan) and frozen in a deep freezer (−80 °C) for at least 30 min. Next, 30 μm-thick sections were cut from the frozen brain block using a cryostat at −15 °C (Leica CM1100, Leica Microsystems, Wetzlar, Germany) and were then mounted on poly-L-lysine-coated glass slides (#S7441, Matsunami Glass Ind., Ltd., Osaka, Japan). Each section was incubated in HistoVT One (#06380-05, Nacalai Tesque, Inc., Kyoto, Japan) at 55 °C for 10 min (repeated twice) to enhance the antigen–antibody reaction. After further incubation for 30 min in PBS containing 0.2% Tween 20 and 10% normal donkey serum (#565-73631, FUJIFILM Wako Pure Chemical Corp.) at room temperature, the sections were incubated overnight at 4 °C in double-antibody solutions containing the following combinations: (1) To detect GFP and CRH, mouse monoclonal antibody for GFP (#M048-3, MBL Life Science, Tokyo, Japan) (1:200) and rabbit polyclonal antibody against CRH (#H-019-06, Phoenix Pharmaceuticals, Burlingame, CA, USA) (1:100); and (2) to detect RFP and CRH, mouse monoclonal antibody for RFP (#M155-3, MBL Life Science) (1:200) and rabbit polyclonal antibody against CRH (#H-019-06) (1:100) as primary antibodies in PBS containing 0.2% Tween 20 and 10% donkey serum. For secondary antibodies to detect the GFP and CRH: an Alexa Fluor 488, donkey anti-mouse IgG (H&L) secondary antibody for GFP (#ab150105, Abcam, Cambridge, UK) (1:200), and an Alexa 555, donkey anti-rabbit IgG H&L (#ab150106, Abcam) (1:200) for CRH, or to detect RFP and CRH, an Alexa Fluor 555, a donkey anti-mouse IgG H&L (#ab150106, Abcam) (1:200) for RFP, and an Alexa 488, a donkey anti-rabbit IgG H&L (#ab150073, Abcam) (1:200) for CRH were used as previously reported ^1^. A confocal scanning laser microscope (LSM 510, Carl Zeiss, Oberkochen, Germany) was used to detect and acquire the fluorescence imaging data. Immunofluorescence was quantified using the Image-Pro Plus imaging software (Media


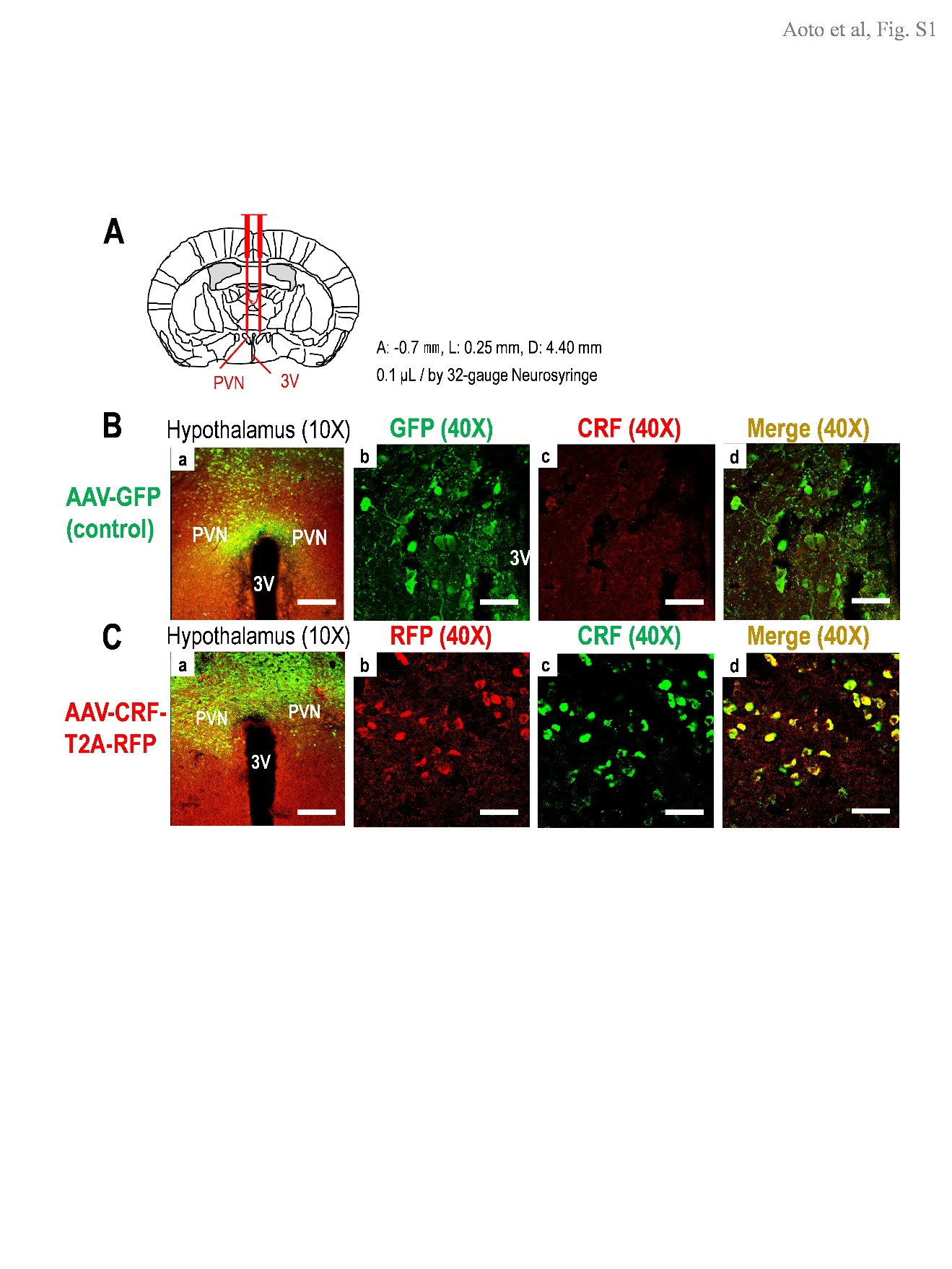
Cybernetics, Silver Springs, MD, USA).

**Supplementary Fig. 1.** (A) AAV-PHP.eB virus injection maps according to the Paxinos mouse brain atlas (anterior: −0.7 mm, lateral: 0.25 mm, depth: 4.40 mm). Red bars around the center of the brain map indicate virus injection needles and the location of virus injections into the hypothalamus, including the PVN (0.1 μL of virus injected by 32-gauge neurosyringe). In the images, “3V” represents the third ventricle and “PVN” represents the paraventricular nucleus of hypothalamus in the red square, in the hypothalamus. (B-C) Transduction of mouse hypothalamus with AAV-PHP.eB vector expressing GFP or RFP and detection of mouse CRH by immunofluorescence in Hy-Crh-control ((a–d); upper lane) in B and overexpression ((a–d); lower lane, Hy-CRH-OE) mice in C. Scale bar = 200 μm (×10 magnification) for (a and e) and 50 μm for (b–d and f–h) (×40 magnification).

**Reference**

1. Kasama E, Moriya M, Kamimura R, Matsuki T, Seki K. Formation of False Context Fear Memory Is Regulated by Hypothalamic Corticotropin-Releasing Factor in Mice. *International journal of molecular sciences*. Jun 3 2022;23(11)doi:10.3390/ijms23116286

2. Challis RC, Ravindra Kumar S, Chan KY, et al. Systemic AAV vectors for widespread and targeted gene delivery in rodents. *Nature protocols*. Feb 2019;14(2):379-414. doi:10.1038/s41596-018-0097-3

3. Williams RW. Mapping genes that modulate mouse brain development: a quantitative genetic approach. *Results and problems in cell differentiation*. 2000;30:21-49. doi:10.1007/978-3-540-48002-0_2

4. De Groot J. *The rat forebrain in stereotaxic coordinates*. North-Holland Publishing Company; 1959.

5. Yang L, Shi LJ, Yu J, Zhang YQ. Activation of protein kinase A in the amygdala modulates anxiety-like behaviors in social defeat exposed mice. *Molecular brain*. Jan 8 2016;9:3. doi:10.1186/s13041-015-0181-3
