# Supplementary figures and images for: Adaptation to Preceding Acute Psychological Stress is Associated With Subsequent Stress Coping Levels via Corticoid Receptors
Source: Alpha Psychiatry. 2025 Aug 26;26(4):46061. doi: 10.31083/AP46061 (PMC12416057; doi:10.31083/AP46061)

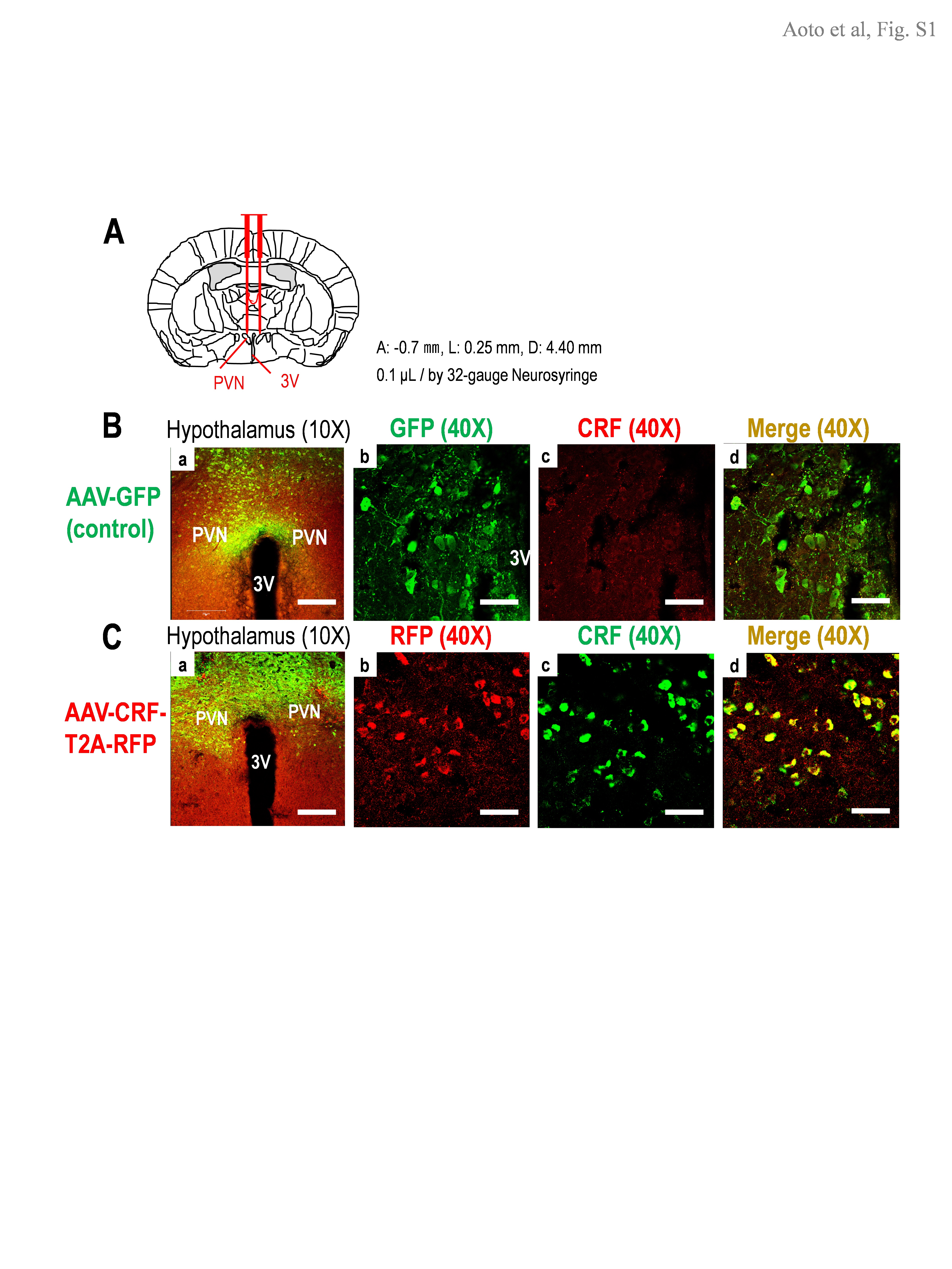

Supplement: Supplementary file 1 [file 2757-8038-26-4-46061-s1.zip › Supplementary Fig. 1.jpg]
